# Parent–infant vocalisations at 12 months predict psychopathology at 7 years

**DOI:** 10.1016/j.ridd.2012.11.024

**Published:** 2013-03

**Authors:** C.S. Allely, D. Purves, A. McConnachie, H. Marwick, P. Johnson, O. Doolin, C. Puckering, J. Golding, C. Gillberg, P. Wilson

**Affiliations:** a Institute of Health and Wellbeing, University of Glasgow, RHSC Yorkhill, Glasgow G3 8SJ, Scotland, United Kingdom; b Robertson Centre for Biostatistics, Boyd Orr Building, University of Glasgow, Glasgow G12 8QQ, Scotland, United Kingdom; c National Centre for Autism Studies at the University of Strathclyde, Glasgow, Scotland, United Kingdom; d Centre for Child and Adolescent Health, School of Social and Community Medicine, University of Bristol, England, United Kingdom; e Centre for Rural Health, University of Aberdeen, The Centre for Health Science, Old Perth Road, Inverness IV2 3JH, Scotland, United Kingdom

**Keywords:** Avon Longitudinal Study of Parents and Children (ALSPAC), Autism, Attention Deficit Hyperactivity Disorder (ADHD), Disruptive behaviour disorders, Vocalisation patterns

## Abstract

This study investigated the utility of adult and infant vocalisation in the prediction of child psychopathology. Families were sampled from the Avon Longitudinal Study of Parents and Children (ALSPAC) birth cohort. Vocalisation patterns were obtained from 180 videos (60 cases and 120 randomly selected sex-matched controls) of parent–infant interactions when infants were one year old. Cases were infants who had been subsequently diagnosed aged seven years, with at least one psychiatric diagnostic categorisation using the Development and Wellbeing Assessment. Psychopathologies included in the case group were disruptive behaviour disorders, oppositional-conduct disorders, Attention Deficit Hyperactivity Disorder, pervasive development disorder, and emotional disorders. Associations between infant and parent vocalisations and later psychiatric diagnoses were investigated. Low frequencies of maternal vocalisation predicted later development of infant psychopathology. A reduction of five vocalisations per minute predicted a 44% (95%CI: 11–94%; *p*-value = 0.006) increase in the odds of an infant being a case. No association was observed between infant vocalisations and overall case status. In sum, altered vocalisation frequency in mother–infant interactions at one year is a potential risk marker for later diagnosis of a range of child psychopathologies.

## Highlights

► Reduced caregiver vocalisations were associated with development of disruptive behaviour disorders. ► Higher infant vocalisations were associated with disruptive behaviour disorders. ► Study into parental vocalisation behaviours early in life may be clinically important.

## 1 Introduction

Early indicators of serious psychopathology exist in the first few years of life and early accurate identification is a prerequisite for early intervention and support (Dawson, 2008). Within parent–child interactions, contingent and mutual responding appears to be crucial in predicting child competence and resilience (Isabella, Belsky, & von Eye, 1989; Tronick, 1989) and may be pivotal in the development of joint attention and language (Gartstein, Crawford, & Robertson, 2007). Infant-directed speech has modified patterns of vocalisation pacing, pitch, content and emphasis. Such components are interpersonally attuned by the adult caregiver to support the infant's attention and engagement as well as to regulate the infant's state and emotions (Marwick & Murray, 2010).

### 1.1 Psychiatric disorders of interpersonal attention and interaction

There are speech and vocalisation indicators that can be used as objective indicators for the diagnosis of hyperactivity syndrome with attention and concentration difficulties. Breznitz (2003) examined the speech and vocalisation patterns of boys (8–10 years) with Attention Deficit Hyperactivity Disorder (ADHD) who were not under the influence of stimulating medication, compared with the speech and vocalisation patterns of boys with reading disabilities and a control group of learners without learning disabilities. Individuals with ADHD were found to speak louder, lack the ability to modulate their voice volume, speak for much longer at a stretch with many short pause durations during their talk but take significantly longer to respond to the conversational partner. To our knowledge, no study has analysed earlier vocal qualities of caregivers and their infants who later go on to receive a diagnosis of ADHD, nor has such vocal analysis been carried out before diagnosis in other psychopathologies such as conduct disorder or other disruptive behaviour disorders.

### 1.2 Vocal expressive behaviours in autism

Children with autism spectrum disorder (ASD) frequently exhibit abnormal voice quality and speech prosody (Bonneh, Levanon, Dean-Pardo, Lossos, & Adini, 2011; Tager-Flusberg, 1981). However, their exact characteristics and underlying mechanisms, as well as their consistency and diagnostic power, remains uncertain (Bonneh et al., 2011; Paul, Augustyn, Klin, & Volkmar, 2005). Despite the potential for enabling an objective and quantitative marker for ASD, current diagnostic criteria do not include atypical vocalisations (Bonneh et al., 2011; DSM-IV, 2000).

### 1.3 A holistic analysis of interpersonal behaviours within early social interaction

Preliminary data from a semi-qualitative analysis by Marwick et al. (2012) recently demonstrated, using a holistic analysis of interpersonal behaviours within early social interaction at one year, that *adult* activity and *adult* speech strongly predict psychiatric diagnosis in the child at seven years of age. Specifically, lower levels of adult activity and lower levels of adult speech significantly predicted caseness and the diagnostic groups of: any ADHD, inattentive ADHD, any emotional disorder, any anxiety disorder, DBDs, oppositional defiant and/or DBD-NOS, and conduct disorder at seven years of age. Analysis of interactive behaviours of the child revealed no such predictors of later psychopathology. To establish the social interactive behaviours that predict later psychiatric diagnosis the authors examined 180 videos of a parent–infant interaction when children were aged one year, from within the Avon Longitudinal Study of Parents and Children (ALSPAC) cohort. Sixty of the videos involved infants who were later diagnosed with a psychiatric disorder at seven years, and 120 were a randomly selected sex-matched control group (Marwick et al., 2012). The analysis was qualitative, impressionistic and examined multiple domains of behaviour (Well-being, Contingent Responsiveness, Cooperativeness, Involvement, Activity, Playfulness, Fussiness and Speech). A Likert scale (from 1 to 5) was used to record the observations. Such scales can be intrinsically insensitive to subtle differences (Finstad, 2010) and Marwick et al.’s (2012) findings of *adult* activity and *adult* speech predicting later psychiatric diagnosis in the child merit further investigation using a more fine-grained, quantitative approach. The reported lack of statistically significant association between later psychopathology and infant vocalisation in Marwick et al.’s paper was surprising and also requires quantitative confirmation or refutation. The approach adopted in the present paper builds on these findings using a robust, quantitative and objective approach to investigating vocalisations in infant–caregiver interactions.

It is also important to explore potential confounding factors or predictors. Marwick et al.’s (2012) results were adjusted either for the sex of the infant alone, or for the sex of the infant and the depression and anxiety scores of the mother at 32–40 weeks gestation and 8 months postnatal. Maternal depression is widely reported to have negative associations with child development and later mental health (Bayley, 1993; Field et al., 2007; Field, Diego, & Hernandez-Reif, 2009; Murray, Kempton, Woolgar, & Hooper, 1993; Murray, Marwick, & Arteche, 2010; Reissland, Shepherd, & Herrera, 2003). In addition to the potential confounders of maternal depression and child gender, we also explored birth weight, and weight, length and ponderal index at 12 months; parental social status (defined by employment type) and the mother's age at the birth of the child. Young maternal age is a risk factor for behaviour problems in children (Wakschlag et al., 2000) and young mothers encounter more adverse family characteristics and, during interactions with their toddlers, demonstrate more inadequate, restrictive and negative parenting practices (D’Onofrio et al., 2009; Trautmann-Villalba, Gerhold, Laucht, & Schmidt, 2004). Preterm birth is a risk factor for psychiatric morbidity in childhood, adolescence and young adulthood (Hack, 2009; Johnson & Marlow, 2011). Increased risk has also been reported in adults born at term with low birth weight, characterised as small for gestational age (SGA), although results have been less consistent (Monfils Gustafsson, Josefsson, Ekholm Selling, & Sydsjo, 2009; Vasiliadis, Buka, Martin, & Gilman, 2010). Low birth weight has also been found to be a risk factor for later development of psychiatric disorders (Lund et al., 2012). Low birth weight predicts increased risk for autism (Burd, Severud, Kerbeshian, & Klug, 1999; Hack et al., 2009), attention-deficit/hyperactivity disorder (ADHD; Hack et al., 2009; Saigal, Pinelli, Hoult, Kim, & Boyle, 2003; Szatmari, Saigal, Rosenbaum, Campbell, & King, 1990; Szatmari, Saigal, Rosenbaum, & Campbell, 1993) and anxiety (Hack et al., 2009; Levy-Schiff et al., 1994). Parental social status has also been found to have an impact on childhood risk for psychiatric disorders (e.g., Gissler, Rahkonen, Järvelin, & Hemminki, 1998; Mäkikyrö et al., 1997).

### 1.4 Present study

Examination of vocal expressive behaviours in early adult–infant interaction would be valuable to enable assessment of the predictive utility of caregiver and infant vocalisation behaviours in relation to later diagnosis of psychopathology. Based on videoed caregiver–infant interactions from a large contemporary birth cohort, we examined whether the quantity and duration of both infant and parent vocalisation could predict child psychopathology at seven years of age. To our knowledge, this is the first study to assess objectively whether the duration and frequency of early vocalisation predicts later psychopathology using a case-control design, nested within a population-based prospective study.

## 2 Method

### 2.1 Participants

The sample consists of participants from the Avon Longitudinal Study of Parents and Children (ALSPAC). ALSPAC is an ongoing population-based study exploring a wide range of environmental and other influences on the health and development of children (Boyd et al., 2012; Golding, Pembrey, Jones, & ALSPAC Study Team, 2001; Golding & ALSPAC Study Team, 2004). Pregnant women resident in the former Avon Health Authority in south-west England, having an estimated date of delivery between 1 April 1991 and 31 December 1992 were invited to take part, producing a ‘core’ cohort of 13,988 singletons/twins alive at 12 months of age. The sample used in the present study consists of an approximately 10% sample of the core ALSPAC cohort who were invited to attend “Children in Focus” clinics after birth. Some 1240 participating families (typically mother/infant dyads) attended the clinic held when infants were 12 months old. A variety of assessments were carried out at the clinic, including the Thorpe Interaction Measure (TIM) (Thorpe, Greenwood, & Rutter, 2003). The TIM involves a caregiver (usually the mother) and their child sharing a picture book, with the caregivers asked to interact with their child in the same way that they would at home. All interactions took place in a ‘living room’ style environment in the clinic, and were videoed. Parents were instructed to stop when the child lost interest in the activity or became distressed and the video recording was terminated.

Our sample comprised 180 videos. Sixty were of infants who were later given a psychiatric diagnostic categorisation of at least one of the following: autism, conduct disorder, attention-deficit/hyperactivity disorder (ADHD), or emotional disorders as assessed using the Development and Wellbeing Assessment (DAWBA) (Goodman, Ford, Richards, Gatward, & Meltzer, 2000) in children remaining in the cohort at 91 months of age. One hundred and twenty videos were randomly selected to form the sex-matched control group. The DAWBA is a structured diagnostic assessment which relies on parental and teacher reports, with final diagnoses confirmed by a child psychiatrist from the questionnaire responses. Diagnostic categories were as follows: disruptive behaviour disorders (ADHD and/or any oppositional/conduct disorder), oppositional-conduct disorders (either conduct disorder, oppositional-defiant disorder or disruptive behaviour disorder-not otherwise specified (DBD-NOS)), ADHD (either combined, inattentive or hyperactive-impulsive type), pervasive developmental disorder (autism), or any emotional disorder (anxiety, depression or phobias). The mother–infant interactions had a mean audio duration of 259 (SD 152) s. Ethical approval was obtained from the ALSPAC Law and Ethics Committee and the Local Research Ethics Committees.

### 2.2 Procedure

Audio files were extracted from the video files, and were used for the analysis. Vocalisations by both the caregiver and the infant were analysed using PRAAT (a free software program for the analysis of vocalisation pitch and timing – http://www.fon.hum.uva.nl/praat/). The software allowed accurate recording of the onset and offset time of each vocalisation. The two raters were blind to the later diagnostic status of the infants.

### 2.3 Statistical methods

Videos with no usable audio were excluded. Due to small numbers, we excluded videos where the sole caregiver was the father. Where both parents were recorded in the video, we present data in relation to maternal vocalisations only.

The total duration of vocalisations (seconds) and the number of discrete vocalisations by both the infant and mother were calculated. By dividing by the total audio duration for each video, this gave the vocalisation rate (total vocalisation duration/total audio duration) and vocalisation frequency (number of vocalisations/total audio duration) for each individual. Twenty randomly selected audio files were appraised by both raters. The reliability of each measure was assessed using inter class correlation coefficients.

Linear regression models were used to assess factors that might be associated with infant and maternal vocalisation rates. Child vocalisation rates were positively skewed and were log transformed prior to analysis. Poisson regression models were used to investigate factors associated with vocalisation frequencies. Models were fitted with total number of vocalisations as the response and the log of the total audio duration as an offset term. Potential predictors were: child gender, birth weight, and weight, length and ponderal index at 12 months; parental social status (defined by employment type), maternal age at birth and depression measured using the Edinburgh Postnatal Depression Scale (EPDS) (Cox, Holden, & Sagovsky, 1987) at 32–40 weeks gestation and at 8 months postnatally. These factors were selected as potential risk factors in infant diagnosis. Variables that were significant at the 10% level were considered potential confounders, and were used to adjust later regression models.

Associations between vocalisation variables and case–control status, and each diagnostic subgroup, were first assessed graphically. Multivariate logistic regression models were fitted to test whether infant and maternal vocalisation variables were independently predictive of diagnostic outcomes after adjusting for potential confounders. In general, vocalisation frequencies demonstrated greater predictive ability than vocalisation rate, so we present results for these variables only. Odds ratios associated with an increase of five vocalisations per minute, with 95% confidence intervals and *p*-values, are presented. Due to the low numbers for some case diagnosis, Firth's penalised-likelihood logistic regression (Firth, 1993) was used.

For the primary analysis of overall case status, a significance level of 5% was used as evidence of an association. The statistical software package R for Windows v2.14 (R Development Core Team, 2012) was used for all analyses and the package logistf (Ploner, Dunkler, Southworth et al., 2010) was used for the Firth's logistic regression.

## 3 Results

No vocalisation data were available for four videos due to poor quality. Seven videos, in which the sole caregiver present was the father, were excluded. The remaining 169 videos from which audio files were extracted included 58 children who went on to develop psychopathology, and 111 controls. Table 1 shows the number of children with any psychiatric diagnosis and within each of the diagnostic subgroups.

Maternal vocalisation rates and frequencies were highly reliable, with interclass correlation coefficients (ICCs) of 83% and 88% respectively. Infant vocalisation rates were slightly less reliable, with an ICC of 61%, but vocalisation frequency was more reliably measured, with an ICC of 85%.

The results of the regression analysis of maternal and infant vocalisation variables are shown in Table 2. Both maternal vocalisation variables were positively associated with the mother's age at birth, and the birth weight of the infant. Child vocalisation rates were predicted by gender, with male children vocalising for longer. Infant vocalisation frequency was predicted by the child's ponderal index and maternal social class, with larger children and children of mothers in non-manual employment having more vocalisations.

**Fig. 1:**
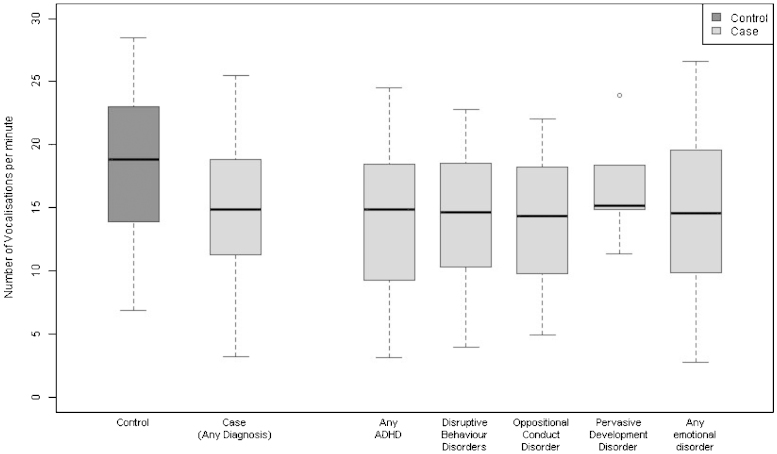
Distribution of the maternal vocalisation frequency amongst controls, cases and each diagnostic subgroup. Each box represents the median and upper/lower quartiles with the whiskers showing the 5th and 95th percentiles.

**Fig. 2:**
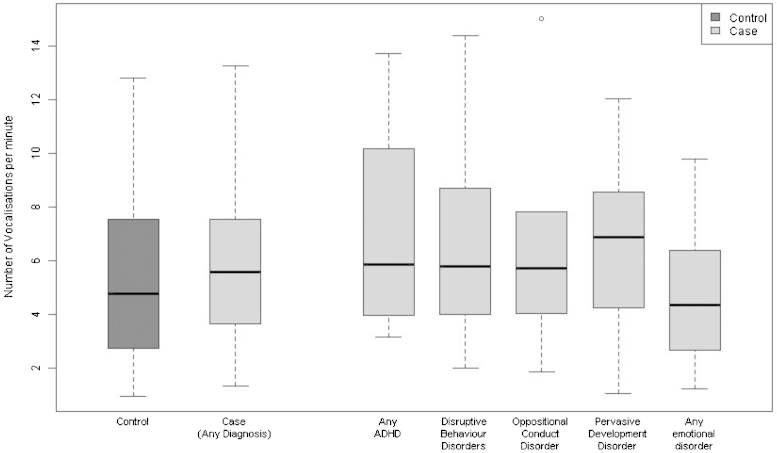
Distribution of the infant vocalisation frequency amongst controls, cases and each diagnostic subgroup. Each box represents the median and upper/lower quartiles with the whiskers showing the 5th and 95th percentiles.

Figs. 1 and 2 show the distribution of infant and maternal vocalisation frequencies per minute amongst controls and cases, and for each diagnostic subgroup. Maternal vocalisation frequency tended to be higher among controls, while child vocalisation frequency was higher among cases.

Table 3 shows the independent associations between child and maternal vocalisation frequencies and later development of psychopathology, adjusted for the potential confounders of child birth weight, child ponderal index, maternal employment category and maternal age at birth, overall and for each diagnostic subgroup. Decreased maternal vocalisation frequency was associated with being a case, with a 31% reduction in the odds of being a case for every increase of five maternal vocalisations per minute. Similar associations were observed for disruptive behaviour disorders, oppositional conduct disorders, emotional disorders and (though not reaching nominal statistical significance) ADHD. Child vocalisation frequency was not associated with overall case status, but did show evidence of being associated with disruptive behaviour disorders, with an estimated 77% increase in the odds of being diagnosed with DBD for every increase of five child vocalisations per minute. Associations of a similar magnitude were observed for oppositional conduct disorders and ADHD, but were not statistically significant. Neither maternal nor child vocalisations were found to predict later diagnosis of pervasive development disorder.

## 4 Discussion

Based on a large cohort of infants, we investigated the predictive utility of parent–child vocalisation at 12 months in the diagnosis of psychiatric disorder. Lower vocalisation frequency in the mother predicted diagnosis of all-case psychopathology in the infant. Subgroup analysis identified a trend in lower maternal frequency of vocalisations being associated with infant diagnoses of disruptive behaviour disorders, oppositional-conduct disorders and emotional disorders. Child vocalisation frequency was not associated with overall case status. Interestingly, higher infant vocalisation frequency *did* show evidence of being associated with disruptive behaviour disorders. Although vocalisation measures were clearly associated with later psychopathology, there are substantial overlaps between case and control values. Considerable refinement will therefore be required before such measures can be used in clinical prediction.

The approach in the present paper enabled the detection of more subtle differences between cases and controls through the use of quantitative, as compared to observational, methods used by Marwick et al. (2012). We report here a difference in vocalisations between case and control infants which was not found by Marwick et al. (2012). The mechanism of this association between increased infant vocalisation and later disruptive behaviour disorders may lie in parental factors such as maternal unresponsiveness (Latimer et al., 2012; Shaw, Bell, & Gilliom, 2000) in genetic factors or gene–environment interactions (Comings, 1997; Faraone, 2004). Similarly, the association between altered maternal vocalisation frequencies and later diagnosis of child psychopathology cannot simply be explained by maternal social class, which has been found in previous research to influence the extent of parental speech to their children (e.g., Hart & Risley, 1995) but confounding by maternal IQ and genetic factors cannot be ruled out in this study design.

Of particular interest were the findings that it was the parents’ vocalisation behaviour that most markedly predicted later onset of psychopathology in the one-year-old infants. Previous studies have found that caregiver behaviour can influence later child psychiatric diagnosis. Adult behaviour during infancy and early childhood can have a negative impact on both child development and later behaviour (Beebe et al., 2011; Belsky, Hsieh, & Crnic, 1998; Olson, Bates, Sandy, & Schilling, 2002). Maternal responsiveness during the first year of life has also been found to be inversely linked to future child conduct problems (Kochanska, 1997; Olson, Bates, Sandy, & Lanthier, 2000; Shaw et al., 2000; Wakschlag & Hans, 1999) and low maternal involvement in play with young children may be an important factor in the development of behaviour problems (Mash & Johnston, 1982). Our finding of reduced frequency of maternal vocalisations being significantly associated with later diagnosis of behaviour disorders in the infant may similarly indicate reduced maternal involvement and responsiveness at one year. Adult speech or involvement has previously been reported to be associated with child psychiatric diagnosis: low maternal involvement in play may be an important factor in the development of behaviour problems (Gardner, 1994). Compared to controls, mothers of children with ADHD were found to initiate less interaction and respond less often to child initiated interaction (Mash & Johnston, 1982). A weakness of this study is that it does not enable a direct investigation of the directionality of effect in the mother-child interaction (Mash & Johnston, 1982).

We did not find that simple infant vocalisation measures predicted autism. One previous study found that 6–12 month old infants at high risk for ASD were found to produce, on average, significantly fewer speech-like vocalisations and more non-speech vocalisation compared to their low risk peers (Paul, Fuerst, Ramsay, Chawarska, & Klin, 2011). Early vocalisation differences in children with autism or at risk of a diagnosis of autism have been found in other studies (Schoen, Paul, & Chawarska, 2011; Warren et al., 2010). A potential explanation for the differences in findings between our study and those of previous studies could be the context of the mother–infant interaction in the present study. However, our sample included only six cases of autism so there may not have been sufficient power to predict caseness. The structure of the observational situation used here reduced the social demand of the context and modulated the child's activity and behaviour, the recording being stopped when the child lost interest or became distressed by the task. Furthermore, we simply examined quantity and did not assess the quality or sequential patterns of vocalisation between mother and child (Stern, 1985/2000; Trevarthen, 2001).

While simple infant vocalisation measures were not found to predict autism in our study, they were found to predict disruptive behaviour disorders. Specifically, increased infant vocalisations predicted disruptive behaviour disorders, which is in contrast to the adult caregivers where *reduced* vocalisations were found to be associated with the subsequent development of disruptive behaviour disorder in the child. To our knowledge, no previous study has investigated earlier vocal qualities of caregivers and their infants who later go on to receive a diagnosis of disruptive behavioural disorder, so this finding suggests that further research into this association may be of clinical importance.

### 4.1 Limitations

A potential limitation of the present study is the relatively small diagnostic sub-group sample sizes, particularly in relation to pervasive developmental disorder. Additionally our analysis focused only on vocalisation duration and frequency. An extended analysis of intonation and content could have been informative but the sound quality on the videos, while adequate for rating occurrence and onset and offset times of vocalisations, precluded more detailed linguistic and pitch contour analysis.

Furthermore, in the present study, there may be some chance of cases being within the control group and vice versa. It is well known that both under-diagnosis and over-diagnosis routinely occur in ADHD (Reid, Hakendorf, & Prosser, 2002).

### 4.2 Strengths of the study

The present study used recorded material collected within a prospective longitudinal community-based cohort study. This allowed analysis of vocalisation patterns in maternal–infant interaction at a very young age to be carried out in relation to a range of later psychiatric diagnostic categorisations, and also avoided the use of retrospective parental reports, which can be biased and subject to recall/memory problems. An additional strength of the present study is that all the children in the study received an independent psychiatric diagnostic categorisation using the DAWBA at age 7 years (Goodman et al., 2000). We also demonstrated that maternal depression at 32–40 weeks gestation and at 8 months postnatally did not account for the observed relationship between vocalisation and later psychopathology. Lastly, our analysis identified and controlled for a number of potential confounding influences upon the associations under consideration.

### 4.3 Clinical implications

Investigating early predictors or risk factors for later childhood psychopathology is important for early detection and timely intervention (Zahn-Waxler, Iannotti, Cummings, & Denham, 1990). The present findings indicate that vocalisation patterns offer an area of potential clinical interest in assessing developmental risk. Our findings tend to support the widely held view that advising mothers to talk more with their children is important for their future development.

### 4.4 Future research

A future prospective cohort study could explore whether vocal elements other than duration and frequency in parent–infant interaction can predict later child psychopathology. The association between later child psychopathology and other elements within caregiver–infant early vocal communicative expressiveness such as rhythmic timing in the alternation of maternal and infant utterances, patterns of intonation, atypical use of pitch and volume modulation could be explored.

We found that reduced levels of caregiver vocalisation and increased levels of infant vocalisation predicted later childhood development of disruptive behaviour disorders. An exploration of the relative effects of genetic and behavioural contributions made by parental ADHD or other neurodevelopmental disorders on the child's psychopathology would be of significant clinical interest.

The next logical step is to analyse synchronicity patterns in the videos, and the relationship of caregiver vocalisation with parenting behaviours. Such analyses should yield useful data on the relationship between broader aspects of the observed parent–infant interaction and the development of psychopathology.

## 5 Conclusion

There is a clear statistical association between reduced vocalisation frequencies in the adult caregiver and subsequent diagnosis of disruptive behaviour disorder, oppositional or conduct disorders, and emotional disorder. By contrast, increased levels of infant vocalisation are associated with disruptive behaviour disorders. Our findings highlight the potential value of analysis of parent–infant vocalisations in predicting later development of psychopathology. It remains to be established whether analyses of this kind can contribute to the development of screening instruments for disorders amenable to early intervention (Wilson, Minnis, Puckering, & Gillberg, 2009).

## Conflicts of interest

The authors declare that they have no conflicts of interest.

## Figures and Tables

**Table 1:** Summary of the number of cases and controls by gender and the number in each subgroup.

	Total	Female	Male
N videos	169	53	116
Controls	111	35	76
Cases	58	18	40
Diagnostic subgroups			
Disruptive behaviour disorders	35	8	27
Any ADHD disorder	16	2	14
Any oppositional-conduct disorder	26	6	20
Pervasive development disorder	6	1	5
Any emotional disorder	27	12	15

**Table 2:** Predictors of infant and maternal vocalisation. Association between selected predictors and child and maternal vocalisation rates and vocalisation frequencies, shown as effect estimates with 90% confidence intervals. Associations in bold were significant at *p* < 0.1.

Predictor variable	Vocalisation rate		Vocalisation frequency	
	Child^a^	Mother^b^	Child^a^	Mother^a^
Child gender (male vs. female)	**1.30 (1.01, 1.66)**	0.60 (−2.58, 3.77)	1.20 (0.92, 1.56)	1.01 (0.85, 1.19)
Child birthweight (kg)	1.16 (0.92, 1.47)	**3.12 (0.18, 6.07)**	1.13 (0.90, 1.43)	**1.17 (1.00, 1.35)**
Child weight (at 12 months (kg))	1.01 (0.91, 1.13)	0.23 (−1.17, 1.62)	1.09 (0.99, 1.20)	1.03 (0.97, 1.10)
Child length (at 12 months (cm))	0.99 (0.94, 1.04)	−0.33 (−0.96, 0.29)	1.01 (0.96, 1.05)	1.00 (0.97, 1.02)
Child ponderal index (at 12 months)	1.02 (0.96, 1.09)	0.65 (−0.16, 1.46)	**1.08 (1.01, 1.15)**	1.04 (0.99, 1.08)
Maternal depression (at 8 months postnatal)	1.00 (0.97, 1.02)	0.12 (−0.17, 0.41)	1.00 (0.98, 1.02)	1.00 (0.99, 1.02)
Maternal depression (at 32–40 weeks gestation)	1.00 (0.98, 1.03)	0.16 (−0.11, 0.43)	0.99 (0.97,1.01),	0.99 (0.97, 1.00)
Mother social class (non-manual vs. manual)	1.24 (0.88, 1.74)	−3.49 (−7.92, 0.95)	**1.65 (1.07, 2.56)**	0.95 (0.76, 1.20)
Father social class (non-manual vs. manual)	0.83 (0.65, 1.07)	−1.38 (−4.6, 1.84)	0.92 (0.71, 1.19)	1.06 (0.90, 1.26)
Mother age at birth (per 5 year increase)	1.03 (0.97, 1.08)	**3.24 (1.63, 4.86)**	1.00 (0.87, 1.13)	**1.09 (1.00, 1.18)**

a Effect estimate represents relative change in vocalisation variable associated each predictor.

b Effect estimate represents absolute change in vocalisation variable associated each predictor.

**Table 3:** Logistic regression analyses of overall case status and diagnostic subgroups, predicted by infant and maternal vocalisation frequencies, adjusted for child birth weight, child ponderal index at 12 months, maternal employment category and maternal age at birth. Effect estimates shown as odds ratio (OR) associated with increases of 5 vocalisations per minute, with 95% confidence interval (CI) and *p*-value.

	OR (95% CI), *p*-value	
	Child vocalisation	Maternal vocalisation
All cases	**1.38 (0.88, 2.19),*****p***** = 0.158**	**0.69 (0.52, 0.90),*****p***** = 0.006**
Diagnostic subgroup		
Any ADHD disorder	1.76 (0.93, 3.41), *p* = 0.084	0.69 (0.43, 1.06), *p* = 0.092
Disruptive behaviour disorders	**1.77 (1.07, 3.05),*****p***** = 0.026**	**0.68 (0.47, 0.94),*****p***** = 0.019**
Any oppositional-conduct disorder	1.69 (0.97, 3.02), *p* = 0.065	**0.64 (0.41, 0.94),*****p***** = 0.023**
Pervasive development disorder	1.30 (0.47, 3.12), *p* = 0.587	0.76 (0.38, 1.36), *p* = 0.408
Any emotional disorder	0.89 (0.42, 1.71), *p* = 0.738	**0.63 (0.42, 0.92),*****p***** = 0.017**
